# Successful Multidisciplinary Treatment with Laparoscopic Hepatectomy and Adjuvant Therapy for Metachronous Solitary Hepatic Metastasis after Excision of a Primary Anorectal Malignant Melanoma: A Case Report

**DOI:** 10.3390/curroncol31010013

**Published:** 2023-12-29

**Authors:** Ryotaro Shimazaki, Masahiro Hagiwara, Chikayoshi Tani, Hiroyoshi Iwata, Hiroyuki Takahashi, Marika Fukuyama, Taisuke Matsuya, Koji Imai, Sayaka Yuzawa, Mishie Tanino, Hideki Yokoo

**Affiliations:** 1 Division of Hepato-Biliary-Pancreatic and Transplant Surgery, Department of Surgery, Asahikawa Medical University, 2-1 Midorigaoka-Higashi, Asahikawa 078-8510, Japan; 2 Division of Gastrointestinal Surgery, Department of Surgery, Asahikawa Medical University, 2-1 Midorigaoka-Higashi, Asahikawa 078-8510, Japan; 3 Department of Dermatology, Asahikawa Medical University, 2-1 Midorigaoka-Higashi, Asahikawa 078-8510, Japantaisuke@asahikawa-med.ac.jp (T.M.); 4 Department of Diagnostic Pathology, Asahikawa Medical University, 2-1 Midorigaoka-Higashi, Asahikawa 078-8510, Japan

**Keywords:** anorectal malignant melanoma oligometastasis, immunotherapy, oligometastasis

## Abstract

Anorectal malignant melanoma (ARMM) is extremely rare and generally lethal, irrespective of the treatment administered. The disease is often diagnosed late, metastases being present in approximately two-thirds of patients at the time of initial diagnosis. Solitary metastasis of ARMM to a distant organ is exceedingly rare. A 76-year-old woman with a history of laparoscopic abdominoperineal resection of an ARMM 13 months previously, was found to have a solitary liver metastasis in the follow-up computed tomography. A preoperative work-up showed no other distant metastases nor contraindication to surgery. It was therefore considered that resection was indicated. The metachronous solitary liver metastasis from an ARMM was treated by laparoscopic wedge hepatectomy of the eighth segment 18 months after excision of her primary ARMM. Adjuvant therapy with pembrolizumab was initiated and continued at 6-week intervals. The patient has not exhibited any immune related Adverse Effects (irAE) during or subsequent to treatment with pembrolizmab and has now completed 12 months of adjuvant pembrolizumab therapy, having survived 33 months from the initial operation for primary ARMM, and remaining recurrence-free 14 months after hepatectomy. ARMM is extremely rare and resection of a metachronous solitary metastasis followed by adjuvant therapy has not previously been reported. We hope this case will be useful for clinicians who might treat similar patients.

## 1. Introduction

Anorectal malignant melanoma (ARMM), an extremely rare and frequently lethal disease, arises predominantly in the mucous epithelium of the antral dentate line in the anus and rectum [1]. ARMM is often diagnosed late because the symptoms are nonspecific, delaying presentation [2]. Metastases are present at the time of initial diagnosis in about 70% of individuals with ARMM, being multiple in more than 80% of cases [3,4]. To the best of our knowledge, only two cases of hepatic resection for a synchronous solitary liver metastasis from a primary ARMM have been reported [3,5]. Additionally, we found only one report of hepatic resection of a metachronous solitary liver metastasis after surgery for primary ARMM [6].

## 2. Case Report

**Figure 1:**
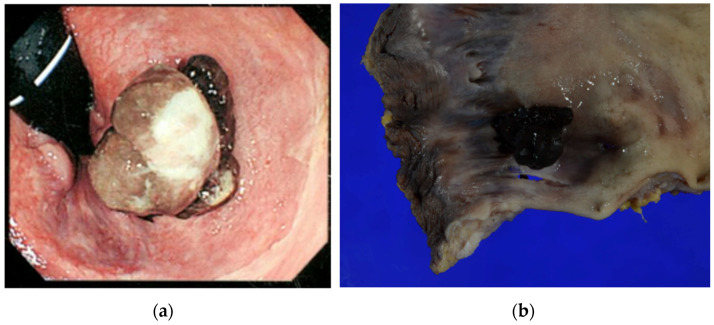
(**a**) Endoscopic findings before the abdominoperineal resection. A polypoid lesion with brownish pigmentation is visible between the dentate line and anal verge. (**b**) Photograph of operative specimen showing a 15 mm × 15 mm polypoid pigmented lesion (15 mm in vertical thickness) on the dentate line.

Our patient, a 69-year-old woman, presented with anorectal discomfort and blood in her stools. Fourteen months before her presentation to our institution, she had visited her general practitioner because of tenesmus and hematochezia. The provisional diagnosis was hemorrhoids, and she was referred to a nearby hospital for colonoscopy, which revealed a protruding black lesion on the antral dentate line that was highly suspicious of an ARMM (Figure 1).

The patient was referred to the Gastrointestinal Surgery Department of the Asahikawa Medical University Hospital for further treatment, where she underwent elective laparoscopic abdominoperineal resection, D3 lymphadenectomy, and transperineal total mesorectal excision one month after the initial presentation. The postoperative pathological diagnosis was anorectal mucosal melanoma, pT4aN0M0, pStageIIB, 15 mm × 15 mm × 15 mm, vertical thickness 15 mm, infiltration into at least the submucosa, no lymphovascular invasion, and resection margin negative for malignancy (R0) in accordance with the NCCN guideline v2.2019 (https://www.nccn.org/guidelines/guidelines-detail?category=1&id=1492, accessed on 27 March 2021) (Figure 1). Postoperatively, she was followed up at 6-month intervals with blood tests and computed tomography (CT). No adjuvant therapy was administered.

**Figure 2:**
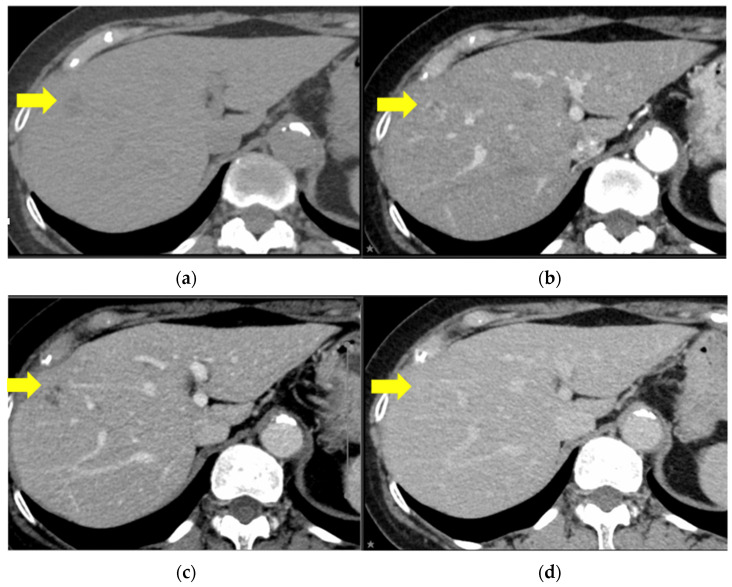
Contrast enhanced computed tomography images. There is a round 27 × 26 mm lesion in S8 with mild ring-enhancement during the arterial phase and an irregularly contrasted hypodense area within the lesion. The yellow arrows indicate the features listed. (**a**) Plain, (**b**) arterial phase, (**c**) portal phase, and (**d**) equilibrium phase.

Thirteen months after the initial surgery, a solitary mass was identified on the surface of the eighth segment of the liver by CT and subsequent magnetic resonance imaging (Figure 2).

**Figure 3:**
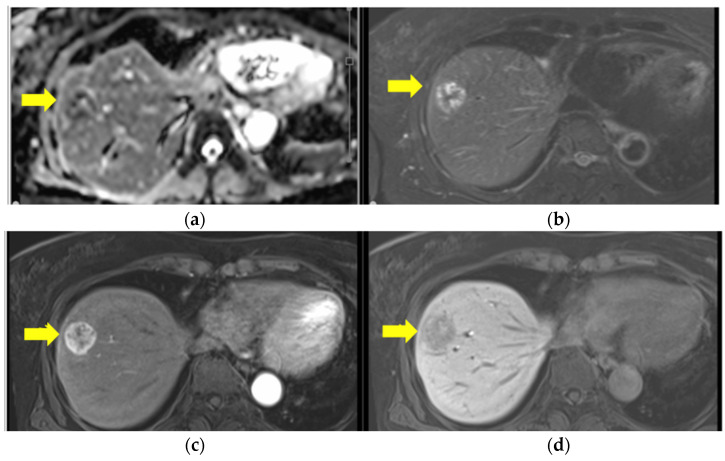
Ethoxibenzyl (EOB) magnetic resonance imaging before hepatectomy. (**a**) T1W1: mixed high and low, (**b**) T2W1: peripherally high signal. There is a well-circumscribed round lesion in S8 with ring enhancement in the EOB-arterial phase (**c**) and a low signal in the EOB-hepatic phase (**d**).

Ultrasound-guided percutaneous needle biopsy of the lesion was performed 2 months later. The histopathological findings were compatible with a diagnosis of a liver metastasis from her primary anorectal malignant melanoma. Initially, the patient did not wish to undergo any further treatment. However, given that the metastasis was solitary and appeared to be confined to the liver, there were no detectable metastases in other locations (brain, lung, gastrointestinal tract or lymph nodes) on fluorodeoxyglucose-positron emission tomography or CT (Figure 3).

**Figure 4:**
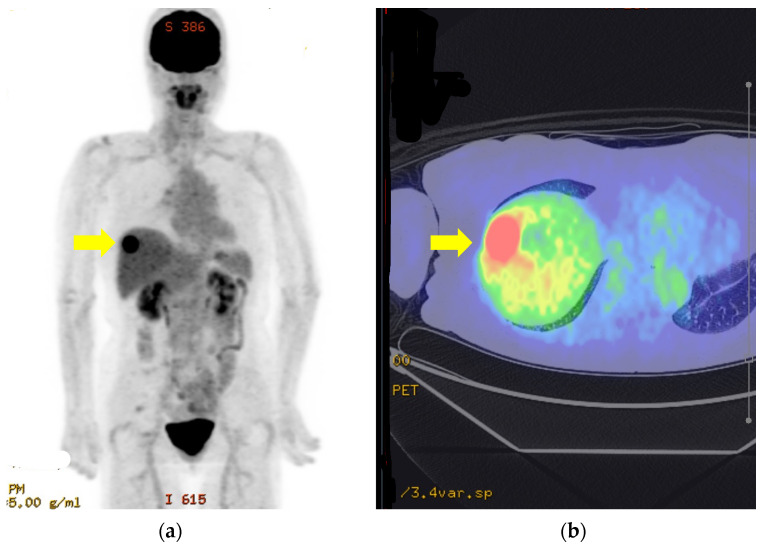
Fluorodeoxyglucose-positron emission tomography images of solitary liver metastasis (**a**,**b**).

Three treatment options were therefore proposed. These comprised systemic therapy with an immune checkpoint inhibitor, radiological ablation with adjuvant therapy, or laparoscopic hepatectomy with adjuvant therapy. She decided to undergo surgical resection with adjuvant therapy and was accordingly referred to the Department of Hepato-biliary-pancreatic and Transplantation surgery for an elective procedure. Her preoperative work-up, including fluorodeoxyglucose-positron emission tomography (Figure 4) and relevant blood tests, yielded no significant findings.

There is a lesion in S8 (yellow arrows) with Max SUV17.8. There is no evidence of metastasis to other organs or lymph nodes (a, b).

**Figure 5:**
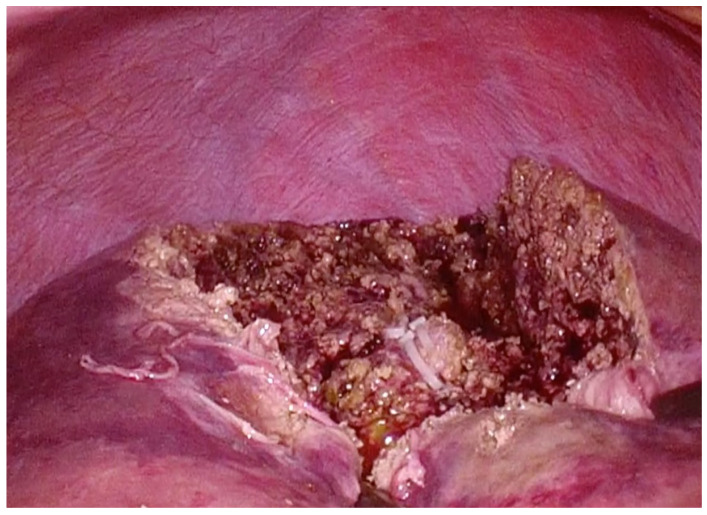
Intraoperative photograph taken immediately after completion of wedge resection of the eighth segment.

Laparoscopic partial hepatectomy of the eighth segment was performed under ultrasonographic guidance 18 months after the initial surgery (Figure 5).

Her postoperative course after hepatic resection was unremarkable; there were no complications. She resumed drinking water on Postoperative Day 1, had her first meal on Postoperative Day 3, and was discharged from hospital on Postoperative Day 10.

**Figure 6:**
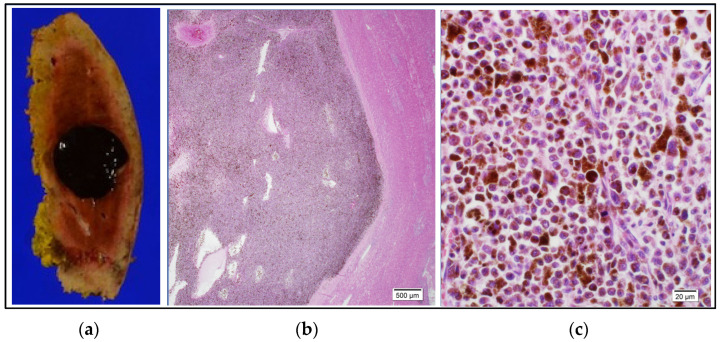
Histopathological findings. (**a**): Photograph of operative specimen showing a 20 mm × 20 mm well-circumscribed, darkly pigmented lesion. The resection margin was negative (R0). (**b**): Photomicrograph of a low power field of a hematoxylin and eosin-stained section showing aggregation of pigmented cells, a distinct border, and normal hepatocytes in the background. (**c**): Photomicrograph of a hematoxylin and eosin-stained high-power field (×40) showing pleomorphic cells of varying size with a high nuclear/cytoplasmic ratio containing melanin pigment in the cytoplasm. Testing for *BRAF* mutation was negative.

The histopathological diagnosis on examination of the operative specimen was metastasis from her anorectal malignant melanoma. The dissection margin was negative (R0) and further examination for *BRAF* mutation was negative (Figure 6).

She was therefore commenced on adjuvant therapy with an immune check point inhibitor, pembrolizumab (400 mg), at 6-weekly intervals. She completed the planned full course of 12 months. Thus far, there has been no evidence of recurrence of disease or immune-related adverse effects.

## 3. Discussion and Conclusions

Anorectal malignant melanoma, a rare and frequently lethal malignancy, was first described by Moore in 1857 [7]. ARMM accounts for 0.38–1% of all anorectal malignancies and 0.25–1.3% of all malignant melanomas. The mean age of diagnosis is 50–70 years. Approximately two-thirds of these lesions are pigmented and most are located within the anal canal or at the anal verge, the remainder originating in the rectum [8,9].

ARMM often presents with nonspecific symptoms, including hematochezia, anal pain, tenesmus, palpable anal mass/swelling, and change in bowel habit, often resulting in delayed diagnosis [4,8,10]. ARMM is sometimes misdiagnosed as thrombosed hemorrhoids, or anal polyps in the case of amelanotic melanomas, which account for approximately 30% of all ARMMs [2,10].

Diagnosis is often delayed in the absence of a high index of suspicion for this condition. There are no specific diagnostic tests for ARMM. Serological markers such as lactate dehydrogenase and 5-S-cystenyldopa are the only relevant serological tests that are widely available in Japan. They can be utilized as biomarkers for evaluation of response to therapy or detection of metastases, but are not sensitive enough for diagnosis [1,11].

On endoscopic examination, ARMMs are characteristically darkly pigmented polypoid lesions. However, between 20% and 30% are completely amelanotic. Therefore, definitive diagnoses require endoscopic biopsy and subsequent histopathological investigation, ideally with immunohistochemical staining for protein-S, HMB-45, and Melan A [12].

ARMMs are characterized histopathologically by pleomorphic cells with high nuclear-cytoplasmic ratios and pigment granules in the cytoplasm. Immunochemical staining is invaluable in diagnosing amelanotic ARMMs.

Delays in diagnosis or presentation at an advanced stage are not uncommon because these lesions may be mistaken for hemorrhoids or adenomatous polyps. Because there is no anorectal melanoma-specific staging system, ARMMs are usually staged according to the American Joint Commission on Cancer guidelines for cutaneous melanoma [1]. Given that advanced stage is an independent prognostic factor for mortality, early diagnosis is crucially important in management of ARMMs.

Surgery is the first line therapy for the approximately one-third of patients who present with localized disease. Surgical treatment by either abdominoperineal resection (APR) or wide local excision (WLE) with curative intent is appropriate in these patients, but not for those with synchronous local or distant metastases at the time of diagnosis [2,12].

APR achieves a lower local recurrence rate and better locoregional control than WLE [13]; however, there is reportedly no statistically significant difference in the 5-year survival rate between these two procedures [13,14,15,16]. The UK national guidelines for ano-uro-genital mucosal melanomas recommend the most conservative excision possible, provided a negative resection margin is achieved. WLE is preferable with respect to comorbidities and restrictions in daily living, whereas APR should be reserved for palliation [14,15]. However, locoregional recurrence rates after WLE are reportedly as high as 50%, often necessitating re-operation (often in the form of APR) that may result in serious morbidity [2]. Kelly et al. have therefore proposed combining local excision with radiotherapy [17].

APR is a reasonable approach in patients without advanced disease because it appears to achieve long-term survival more often than less radical procedures. In Kelly et al.’s series, nine of ten long-term survivors had undergone APR.

A solitary metachronous liver metastasis from ARMM is so rare that we were unable to find any reports of a resection of one. However, two prior reports describing resection of synchronous liver metastases from ARMM have been published [3,5]. Recently, Li et al. published the results of a prospective follow-up and the natural history of 766 cases of membranous melanoma, 188 of which were in the lower gastrointestinal tract (anorectal and colonic). Only one of these patients (0.53%) had a synchronous solitary liver metastasis [4]. The rarity of solitary liver metastasis means there is insufficient information to determine what comprises optimal management.

In recent years, resection of solitary metastases from cutaneous malignant melanoma has reportedly achieved durable long-term survival; thus, one can extrapolate that resection of a solitary metastasis from an ARMM may also be beneficial [18,19].

We believe that solitary lesions should be resected, whereas multiple metastases should be treated with either immune check points (nivolubmab or pembrolizumab) or BRAF/MEK inhibitors in accordance with the guidelines for cutaneous melanomas.

Various new drugs have recently been validated for treatment of cutaneous malignant melanoma, including combinations of molecular targeted agents BRAF, MEK, and immune checkpoint inhibitors such as anti- programmed death 1 and antibodies to anti- cytotoxic T-lymphocyte-associated antigen. These treatments have also been validated as adjuvant therapy in patients at high risk of recurrence [20].

Optimal treatment of ARMM differs according to *BRAF* mutation status. It should be noted that mutations in *BRAF* and *c-Kit* occur infrequently, in approximately 13% and 10% of cases, respectively [4]. If *BRAF* mutation has occurred, molecular targeting drugs such as BRAF and MEK inhibitors can be utilized. When BRAF mutation testing is negative, immune check point inhibitors such as PDL1 or CTLA4 can be used.

Although, the novel therapy with immune check point inhibitors seems promising, there are several disadvantages to the use of immune check point inhibitors, as they may elicit serious immune related Adverse Effects (irAEs), such as pancytopenia, interstitial pneumonia, and autoimmune carditis. Therefore, cautious monitoring for possible occurrence of irAEs is important during the use of immune checkpoint inhibitors, as in pembrolizumab.

In the present case, BRAF mutation testing was negative, therefore pembrolizumab (400 mg) was indicated and administered as adjuvant therapy, the planned 12 months of therapy being completed without recurrence. Fortunately, none of the immune related Adverse Effects have been observed during and after the treatment with 12 months of pembrolizumab in this patient.

Malignant melanoma of the rectosigmoid region is very rare and has a very poor prognosis. Because of its rarity, there is insufficient evidence to establish the optimal treatment strategy. To the best of our knowledge, we here report the first case of successful multidisciplinary treatment comprising surgery and 12 months of adjuvant therapy for a metachronous solitary liver metastasis in a patient who had undergone resection of an ARMM. The patient is currently alive and recurrence-free 33 months after the initial surgery and 14 months after undergoing hepatic metastatectomy.

Recent advances in development of immune checkpoint inhibitors and cumulative knowledge on favorable outcomes of liver metastatactomy for solitary liver metastasis of cutaneous malignant made formulation of the novel multidisciplinary management in this case. Our findings suggest that resection of metastasis together with adjuvant therapy with pembrolizumab may be feasible option for patients with metachronous solitary liver metastases after surgery for a primary ARMMM. We hope this case will be useful for clinicians who may encounter similar cases.

## Data Availability

Data sharing is not applicable to this article as no datasets were generated or analyzed during the current study.
